# Machine learning-assisted novel recyclable flexible triboelectric nanogenerators for intelligent motion

**DOI:** 10.1016/j.isci.2024.109615

**Published:** 2024-03-29

**Authors:** Yuzhang Wen, Fengxin Sun, Zhenning Xie, Mengqi Zhang, Zida An, Bing Liu, Yuning Sun, Fei Wang, Yupeng Mao

**Affiliations:** 1 Physical Education Department, Northeastern University, Shenyang 110819, China; 2 Faculty of Robot Science and Engineering, Northeastern University, Shenyang 110819, China; 3 Criminal Investigation Police University of China, Shenyang 110035, China; 4 School of Strength and Conditioning Training, Beijing Sport University, Beijing 100084, China

**Keywords:** Health sciences, Physics, Computer science, Materials science

## Abstract

In the smart era, big data analysis based on sensor units is important in intelligent motion. In this study, a dance sports and injury monitoring system (DIMS) based on a recyclable flexible triboelectric nanogenerator (RF-TENG) sensor module, a data processing hardware module, and an upper computer intelligent analysis module are developed to promote intelligent motion. The resultant RF-TENG exhibits an ultra-fast response time of 17 ms, coupled with robust stability demonstrated over 4200 operational cycles, with 6% variation in output voltage. The DIMS enables immersive training by providing visual feedback on sports status and interacting with virtual games. Combined with machine learning (K-nearest neighbor), good classification results are achieved for ground-jumping techniques. In addition, it shows some potential in sports injury prediction (i.e., ankle sprains, knee hyperextension). Overall, the sensing system designed in this study has broad prospects for future applications in intelligent motion and healthcare.

## Highlights

• Waste tissues used for green and sustainable development
• The sensor’s durability and stability enable versatile applications
• The DIMS can achieve intelligent monitoring of sports and injury
• Machine learning assisted sports monitoring empower intelligent motion

## Graphical abstract

**Figure:**
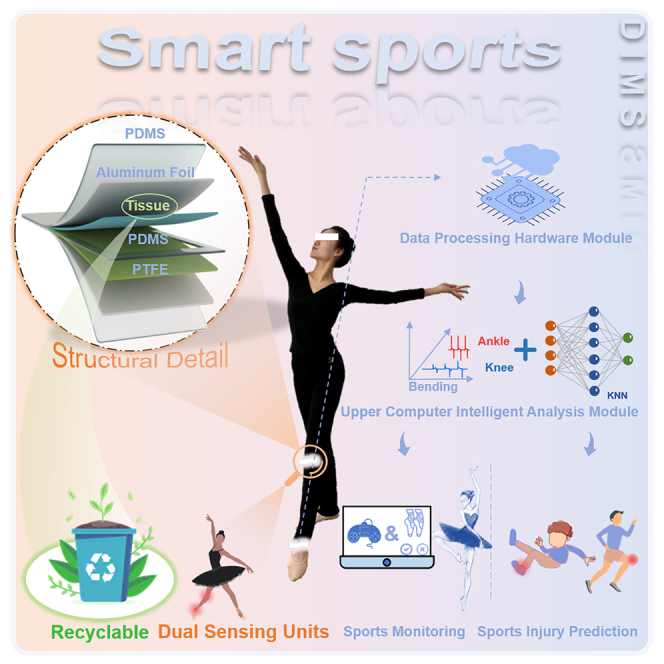

## Introduction

With the development of the Internet and the advent of the smart era, wearable electronic devices have become a focal point of research in various domains, including the Internet of Things, health monitoring, and sports monitoring.^1^^,^^2^^,^^3^^,^^4^^,^^5^ These devices can either be implanted inside the human body or worn on the body’s surface, including smart bracelets, smart watches, smart clothing, etc.^6^^,^^7^^,^^8^^,^^9^ They incorporate built-in sensors to capture the user’s physiological metrics and motion data, which are subsequently processed, analyzed and presented using various algorithms.^10^ However, these devices face several challenges, such as discomfort for users, limitations in portability, limited battery life, reliance on external power sources leading to increased production costs, and environmental concerns.^11^^,^^12^^,^^13^^,^^14^^,^^15^ Additionally, privacy issues arise due to the collection of users’ personal physiological information and motion data.^16^^,^^17^ To address these challenges, it is imperative to develop new sensing technologies that fulfill monitoring requirements, reduce usage costs, enhance device sustainability, and mitigate environmental impact.

Triboelectric nanogenerators (TENGs), as a self-powered technology for efficient energy harvesting and supply, have the advantage of diverse material choices,^18^^,^^19^^,^^20^^,^^21^^,^^22^^,^^23^^,^^24^^,^^25^ enabling a wide range of potential applications across various fields.^10^^,^^26^^,^^27^^,^^28^^,^^29^^,^^30^^,^^31^^,^^32^^,^^33^^,^^34^^,^^35^ Specifically, the utilization of recyclable materials can lower the manufacturing costs of TENGs, and these materials can be reclaimed and reused, thereby minimizing waste generation and promoting sustainability for widespread TENG adoption in wearable systems.^36^^,^^37^ In the realm of motion monitoring, researchers have made successful strides in creating TENGs using recyclable, degradable, and cost-effective materials. For example, some researchers have used materials such as cellulose filter paper and discarded textiles to prepare TENG for human motion monitoring and sweat analysis.^38^^,^^39^^,^^40^ These recyclable materials not only have environmental friendliness but also offer cost advantages compared to pricier materials like polyurethane and stretchable fibers.^41^^,^^42^ In addition, disposable tissues are more widely used in daily life because of their strong antimicrobial properties, ease of use, and cleanliness.^43^ This leads to an increasing number of discarded tissues. Therefore, recycling discarded tissues as triboelectric layer material for the preparation of TENG is important to reduce the environmental impact and solve energy challenges. The combination of recyclable materials and TENGs not only takes full advantage of their energy harvesting and supply but also enhances their environmentally friendly and sustainable characteristics, bringing additional benefits to the development of wearable systems and other domains.

The intelligent integration of TENG technology with diverse fields greatly enriches our lives.^44^^,^^45^^,^^46^^,^^47^^,^^48^^,^^49^ It combines real-time sensing, signal processing, and machine learning technologies to obtain information about the user’s physiological parameters, exercise status, etc., which is subsequently transmitted to intelligent terminals for processing and analysis, significantly improving signal recognition accuracy.^50^^,^^51^ This opens up myriad application opportunities in motion monitoring and medical health. According to the World Health Organization (WHO), approximately 1.71 billion people suffer from musculoskeletal muscle disorders, and osteoarthritis and injuries account for up to 36% of cases.^52^ Knee and ankle pain rank among the primary causes of musculoskeletal disease burden, followed by low back pain. During the dance ground-jumping training, knee and ankle joints are prone to injury.^53^^,^^54^^,^^55^ Dancers repeat a single motion for a long time, and decreased limb strength and body control, lead to sports injuries.^56^^,^^57^^,^^58^ High-intensity counter-joint motion can cause irreversible damage to the lower extremities, especially the knee.^59^ In addition, daily activities, and sports can lead to ankle injuries.^60^^,^^61^^,^^62^ Therefore, it is urgent to develop an intelligent motion monitoring system based on TENG technology to track sports performance and prevent sports-related injuries.

In this work, a recyclable flexible triboelectric nanogenerator (RF-TENG) was developed. Specifically, the disposable recyclable tissue paper (wood fiber) and polytetrafluoroethylene (PTFE) film served as the triboelectric layers for RF-TENG, while aluminum foil was employed as electrodes. The outermost layer of RF-TENG was encapsulated in polydimethylsiloxane (PDMS), imparting flexibility and biocompatibility, rendering it suitable for attachment to the skin surface. The resulting RF-TENG achieved real-time sports data transmission with a response time of 17 ms and a recovery time of 26 ms. Remarkably, even after 4200 operations, the proposed RF-TENG exhibited only a 6% variance in output voltage. After 15 and 30 days, the RF-TENG maintained 96% and 93% of its output voltage, demonstrating excellent durability and stability. Additionally, a dance sports and injury monitoring system (DIMS) was designed to identify sports information and provide feedback. Employing the K-nearest neighbor (KNN) algorithm, three classes of dance sports techniques were successfully identified and classified with training and testing accuracies of 97.3% and 98.1%, respectively. This work provides a feasible platform for the application of flexible sensors in the fields of motion assistance and intelligent motion.

## Results and discussion

### Concept and design of a dance sports and injury monitoring system

**Figure 1:**
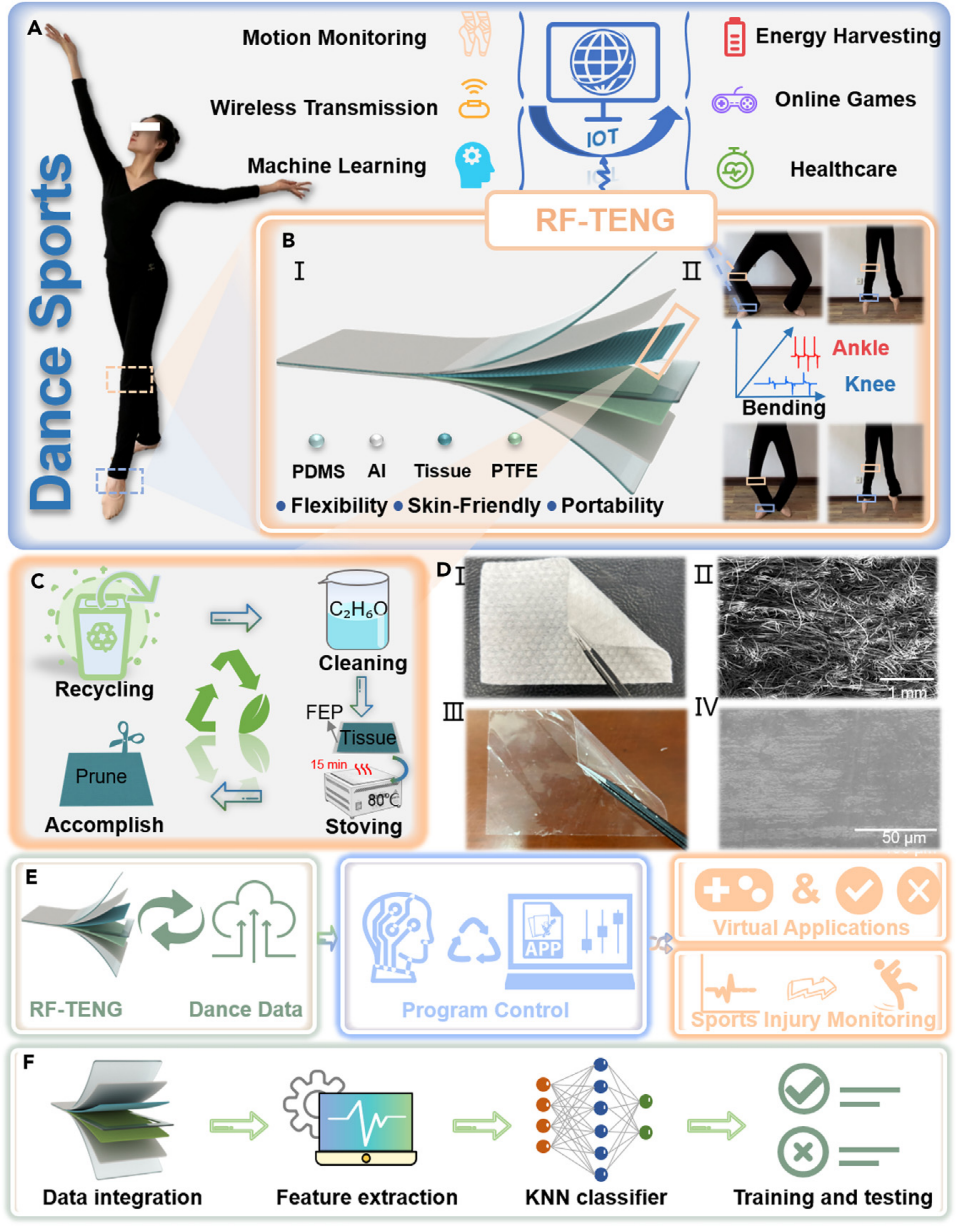
The concept, structure, and design of a dance sports and injury monitoring system (A) Concept diagram of dance sports injury monitoring system. (B) Structure diagram and practical application diagram of RF-TENG. (Ⅰ) Structure diagram of RF-TENG. (Ⅱ) Practical application diagram of RF-TENG. (C) Recycling and preparation process of disposable wood fiber tissue. (D) Optical images, surface electron micrographs (SEM) of the tissue and PTFE. (Ⅰ) Optical image of the tissue surface under bending state. (Ⅱ) SEM of the tissue surface. (Ⅲ) Optical image of PTFE film surface under bending state. (Ⅳ) SEM of the PTFE film surface. (E) Functional flow diagram of the dance sports and injury monitoring system. (F) Data processing flowchart based on KNN.

The concept, structure, and design of a DIMS are shown in Figure 1. The core of the system consists of an RF-TENG sensor module, a data processing hardware module, and an upper computer intelligence analysis module. Figure 1A shows a conceptual diagram of the DIMS. The sports and injury information of the dancer or crowd is collected by the RF-TENG sensor module. The data processing hardware module wirelessly transmits these biosensing signals in real time to the upper computer intelligent analysis module for virtual applications, such as intelligent judgment and feedback and virtual game control. This system meets the monitoring and entertainment needs of daily dance training and monitors the occurrence of some sports injuries. Figure 1B shows the structural diagram and practical application diagram of RF-TENG. Figure 1BⅠ shows the structural diagram of RF-TENG. The RF-TENG consists of a pair of PDMS as an encapsulation layer, a pair of aluminum foil electrodes, PDMS as a support layer, a disposable wood fiber tissue, and PTFE film as a triboelectric layer. Figure 1BⅡ shows the practical application diagram of RF-TENG. The flexible RF-TENG is attached to the knee and ankle joints of the human body for collecting mechanical movement signals. Meanwhile, the RF-TENG minimizes any binding or impact on the dancer’s skin during sports due to its good flexibility. Figure 1C shows the process of recycling and preparation of disposable face tissues. The recovered back tissues are cleaned and dried with anhydrous ethanol to obtain a positive triboelectric layer. (See “method details” section for detailed operations). Figure 1D shows optical images, surface electron micrographs (SEM) of the tissue and PTFE. Figure 1DⅠ shows the surface optical image of a wood fiber tissue with good flexibility. The SEM of the recovered wood fiber is shown in Figure 1DⅡ. Wood fiber disposable tissue has a rough surface and a messy arrangement of wood fibers. Figure 1DⅢ shows the transparency and outstanding flexibility of the PTFE film. And its surface is smooth, as shown in Figure 1DⅣ. Figure 1E shows the functional flow diagram of the dance sports and injury system. The RF-TENG sensor module collects and transmits real-time sports and biological information. The data processing hardware module performs data processing analysis and the upper computer intelligent analysis module realizes virtual applications and sports injury analysis. Figure 1F shows the data processing flowchart based on KNN. Firstly, the sports data of big, medium, and small jumps of ground-jumping techniques are integrated and data feature extraction is performed. Secondly, KNN is utilized for big, medium, and small jump sports techniques recognition and finally, the sports data training and testing accuracy is derived.

### Preparation and working mechanism of RF-TENG

**Figure 2:**
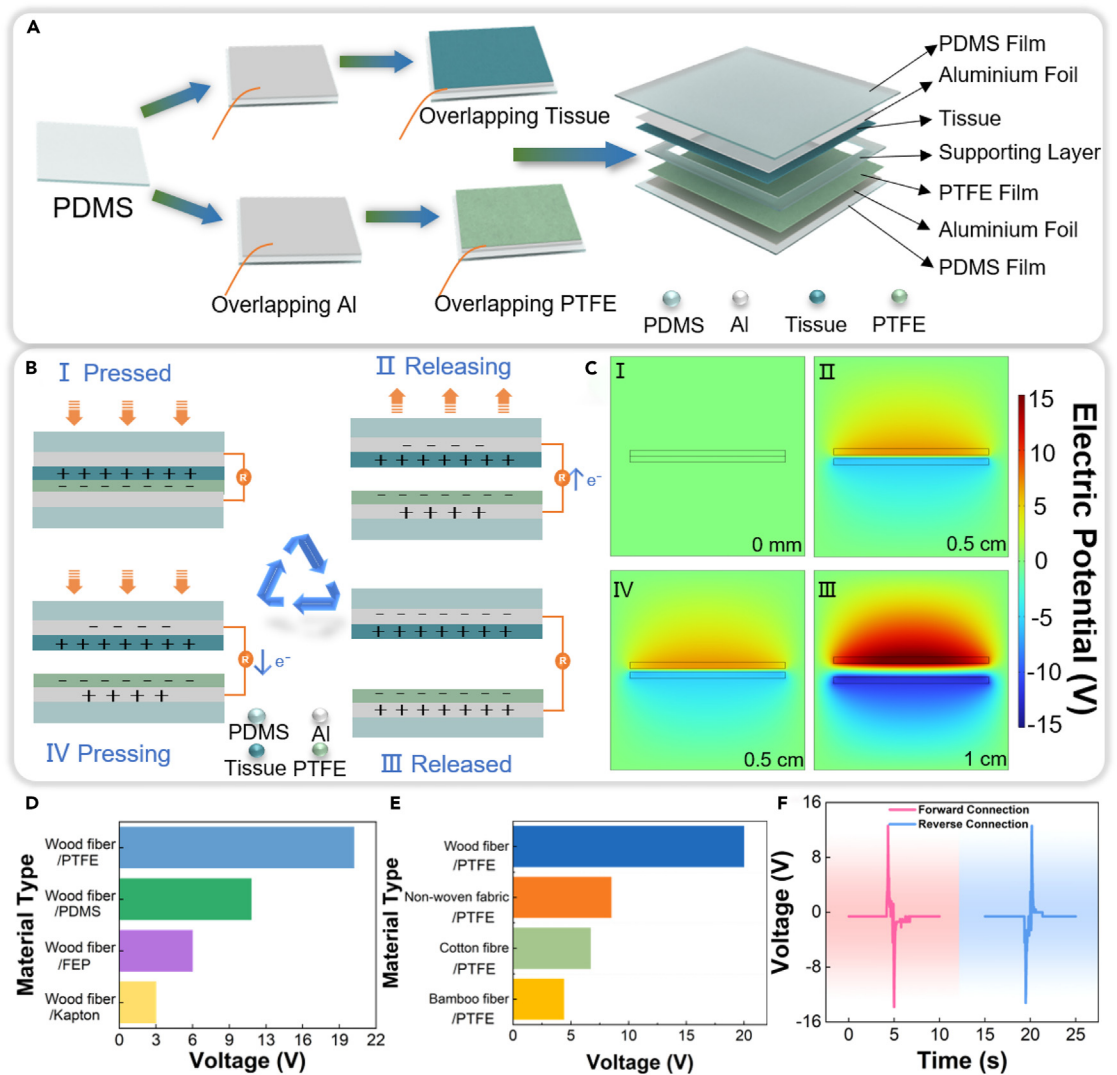
The preparation, working mechanism, simulated potential, and material selection of RF-TENG (A) The preparation process of RF-TENG. (B) The working mechanism of RF-TENG. (C) The potential distribution of RF-TENG is simulated by COMSOL Multiphysics software. (D) The output voltage is generated by the contact separation of wood fiber tissue and different negative triboelectric layers. (E) The output voltage is generated by the contact separation of PTFE film and different material composition tissues. (F) Switching polarity test of RF-TENG.

The preparation process and working mechanism of RF-TENG are shown in Figure 2. Figure 2A shows the preparation process of RF-TENG. Firstly, the PDMS encapsulation layer and PDMS support layer are prepared. Secondly, the aluminum foil electrodes are attached to the surface of the trimmed wood fiber tissue and PTFE film, respectively. Finally, RF-TENG is encapsulated by using PDMS film. (See the “method details” section for a detailed preparation process). It is worth noting that PDMS serves as a support layer with three important roles. Firstly, the hollow structure in the middle of PDMS as a support layer is conducive to the contact separation of the triboelectric layer. Secondly, the inherent flexibility of PDMS ensures that it does not impact the sensor’s flexibility. Finally, the support layer makes the peak characteristics more obvious, which is conducive to the analysis of the motion data. Figure 2B shows the working mechanism of the RF-TENG contact separation mode based on contact electrification and electrostatic induction. When no external force is applied, the wood fiber tissue does not come into contact with the PTFE film. In phase Ⅰ, since the tissue and PTFE film possess different triboelectric sequences, the tissue possesses a strong ability to gain electrons while the PTFE film has a strong ability to lose electrons. When the external force is applied, the two triboelectric layer surfaces generate charges of equal size and opposite polarity, respectively. The charge reaches an equilibrium state and no current is generated in the external circuit, as shown in Figure 2BⅠ. In phase Ⅱ, when the external force is released, the relative separation between the tissue and the PTFE film is achieved. Due to the electrostatic induction effect, electrons are driven to flow from the PTFE film electrode to the tissue electrode, and the external circuit generates a downward current, as shown in Figure 2BⅡ. In phase Ⅲ, the external force is completely released, the separation distance between the PTFE film and the tissue reaches the maximum, the charge reaches the equilibrium state and the external current disappears, as shown in Figure 2BⅢ. In phase Ⅳ, when the external force is applied again, the distance between the tissue and the PTFE film decreases again and the electrons are driven to flow from the tissue electrode to the PTFE film electrode, and the external circuit generates an upward current, as shown in Figure 2BⅣ. To better understand the triboelectric properties of RF-TENG, potential simulations are conducted using COMSOL Multiphysics software, and the results are shown in Figure 2C. Considering the different triboelectric materials with different dielectric constants, the output voltage generated by different frictional pairs is tested. Figure 2D shows the output voltage generated by the wood fiber tissue with different negative triboelectric layers (PTFE, PDMS, fluorinated ethylene propylene (FEP), Kapton). Figure 2E shows the voltage generated by different materials of tissues (wood fiber, non-woven fabric, cotton fiber, bamboo fiber) and PTFE film. Among them, the highest output voltage of 20 V is generated by the frictional pairs of the wood fiber tissue and the PTFE film. Therefore, this article uses this frictional pair as a dielectric layer. Figure 2F shows the voltage waveforms generated by pressing and releasing the RF-TENG in different connection states. It is verified that the RF-TENG emitted the signal, not the measurement system. This provides a prerequisite for the monitoring of periodic movements.

### Sensing properties of RF-TENG

**Figure 3:**
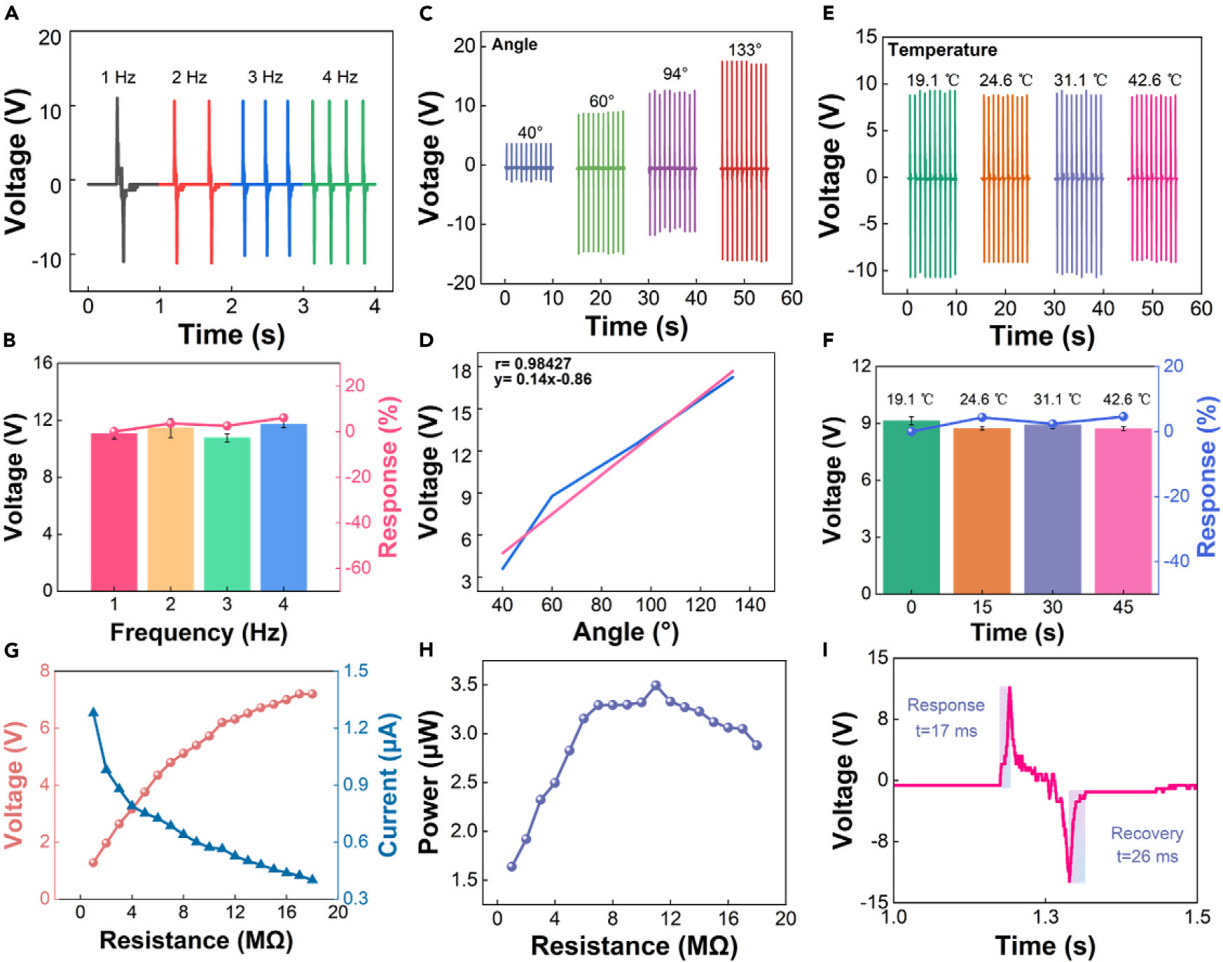
RF-TENG sensing properties testing (A) Output voltage of RF-TENG at different frequencies. (B) Output voltage and response of RF-TENG at different frequencies. (C) Output voltage of RF-TENG at different angles. (D) Linear fitting of RF-TENG output voltage at different angles. (E) Output voltage of RF-TENG at different temperatures. (F) Output voltage and response of RF-TENG at different temperatures. (G) Output voltage and current of RF-TENG at different load resistances. (H) Output power of RF-TENG at different load resistance. (I) Response and recovery time of single press RF-TENG.

The excellent flexibility of the RF-TENG allows it to fit freely in the human knee and ankle joints for joint angle monitoring. To ensure that the RF-TENG can meet the needs of dancers’ sports and injury monitoring, linear motors are used to simulate the human movement state. Figure 3 shows the RF-TENG sensing properties. Figure 3A shows the output voltage of RF-TENG under different frequency conditions (See Figure S1 for the complete diagram). When the frequency is 1 Hz, 2 Hz, 3 Hz, and 4 Hz, the average output voltage of RF-TENG is 11.04 V, 11.44 V, 10.76 V, and 11.71 V, respectively. This indicates that the RF-TENG has excellent sensitivity and stability in the range of human movement frequencies. To more clearly express the stability of the RF-TENG output voltage under different frequency conditions, the response of the RF-TENG under different frequency conditions is calculated, as shown in Figure 3B. The response of voltage can be calculated by the following equation:

(Equation 1) $$R\%=\left|\frac{V_{i}-V_{0}}{V_{0}}\right|\times 100\%$$

Where *V*_*0*_ is the output voltage at 1 Hz and *V*_*i*_ is the output voltage at other frequencies. When the frequency is 1 Hz, 2 Hz, 3 Hz, and 4 Hz, the response of RF-TENG output voltage is 0%, 4%, 3%, and 6%, respectively. It can be seen that the output voltage of the RF-TENG has excellent stability and provides monitoring conditions for cyclic movement technique actions. A simple test bench is designed to measure the output voltage of RF-TENG at different angles, and its schematic diagram is shown in Figure S2. The length “L” of the triangle represents the half of the actual length of RF-TENG. The height “h” of the triangle represents the displacement distance of the linear motor (for every 0.1 increase in the absolute motion value in a linear motor system, the displacement distance “h” increases by 0.6 cm), and the value of “h” is obtained by setting different absolute motion values. According to the cosine function, the RF-TENG angle is calculated (2cosα). 180° - 2Cosα represents the bending angle of the sensor. Figure 3C shows the output voltage of RF-TENG under different angle conditions. When the bending angle is 40°, 60°, 94°, and 133°, the average output voltage of RF-TENG is 3.6 V, 8.78 V, 12.5 V, and 17.25 V, respectively. The response of RF-TENG under different angle conditions is also calculated as shown in Figure S3, where *V*_*0*_ is the output voltage at 30° and *V*_*i*_ is the output voltage at other bending angles. When the bending angle is 40°, 60°, 94°, and 133°, the response of RF-TENG output voltage is 0%, 144%, 247%, and 379%, respectively. To explore the relationship between RF-TENG bending angle and output voltage, linear fitting is calculated, as shown in Figure 3D. The calculation equation (automatically generated through Origin software) is:

(Equation 2) y=0.14x−0.86

Where y is the output voltage (V), and x is the bending angle (°). The Pearson correlation coefficient of the linearity value r = 0.98427. The good linear relationship between RF-TENG bending angle and output voltage has been verified. The bending angle of the human joint can be calculated based on the output voltage. It can be seen that the reduced control of the knee and ankle joints due to the decreased lower limb strength of the dancer during exercise can be monitored by the RF-TENG. To better demonstrate the sensitivity of RF-TENG, the elastic sphere and an ultra-light aluminum foil are used for testing, as shown in Figures S4 and S5. The results showed that RF-TENG can monitor the fall of the aluminum foil and the air squeezed out by the elastic sphere, demonstrating the good sensitivity of the RF-TENG. To verify the effect of skin temperature on the voltage output of RF-TENG, the output voltage of RF-TENG is tested at different temperatures, as shown in Figure 3E. When the temperature is 19.1°C, 24.6°C, 31.1°C, and 42.6°C, the average output voltage of RF-TENG is 9.14 V, 8.74 V, 8.92 V, and 8.72 V, respectively. To better evaluate the effect of temperature on the output voltage of RF-TENG, its response has also been calculated, as shown in Figure 3F. Where *V*_*0*_ is the output voltage at 19.1°C, and *V*_*i*_ is the output voltage under other temperature conditions. When the temperature is 19.1°C, 24.6°C, 31.1°C, and 42.6°C, the response of RF-TENG output voltage is 0%, 4%, 2%, and 5%, respectively. It can be seen that RF-TENG has excellent thermal stability. Considering that the average temperature of the human skin is 33.8°C, the output voltage of RF-TENG will not be affected when the skin comes into contact with it. Figure 3G shows the output voltage and current of RF-TENG under different loading resistance conditions. According to Ohm’s law, the current and power are calculated by external load resistance. Under load resistance conditions of 0–18 MΩ, the maximum output voltage and current of RF-TENG are 7.2 V and 1.28 μA, respectively. When the load resistance is 11 MΩ, the maximum output power of RF-TENG is 3.49 mW (Figure 3H). Therefore, the internal resistance of RF-TENG is 11 MΩ. Considering the repeatability and duration of sports, the durability (Figure S6) and stability (Figure S7) of RF-TENG were tested. Figure S6 shows the output voltage of the RF-TENG after 4200 cycles of continuous operation, and it is stabilized at 14 V Figure S7 shows that after 15 days and 30 days of resting, the RF-TENG still maintains 96% and 93% output voltage. Its excellent durability and stability are verified to meet the needs of dancers’ daily training. Figure 3I shows the response and recovery time of the RF-TENG with a single press. The 17 ms response time and 26 ms recovery time ensure real-time, efficient transmission of RF-TENG sensor module data. It also provides a guarantee for the application of the upper computer intelligent analysis module. Also, the high sensitivity performance of the RF-TENG is demonstrated by comparison with other literature (Table S1). In summary, RF-TENG has excellent sensing properties and can be able to meet the needs of dancers’ sports and injury monitoring. Meanwhile, it has good application prospects in the field of flexible electronics.

### Energy harvesting properties of RF-TENG

**Figure 4:**
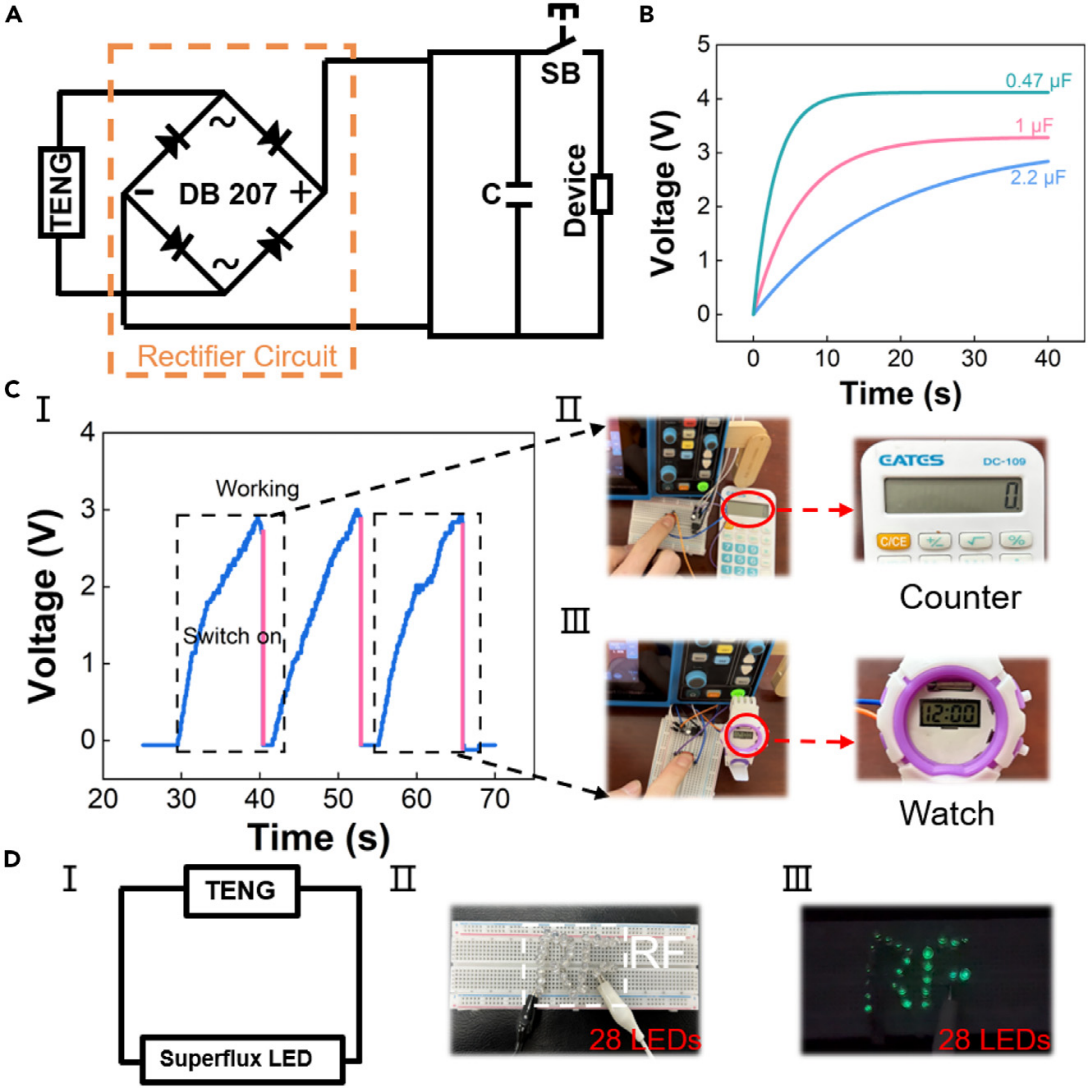
RF-TENG is demonstrated as a power supply for various applications (A) Circuit diagram of RF-TENG supplying power to the device by charging capacitors. (B) Charging curves of RF-TENG for different capacitors. (C) RF-TENG utilizes the power collected by capacitors to drive small calculators and watches. (Ⅰ) Charging and discharging curves for supplying power to the device. (Ⅱ) Optical diagram of the device after driving. (D) Circuit diagram and optical image of RF-TENG lighting up 28 commercial green LEDs by tapping. (Ⅰ) Circuit diagram of RF-TENG for lighting LED. (Ⅱ) The letters “RF” are composed of 28 LEDs. (Ⅲ) Tap RF-TENG to light up the letters “RF”.

Figure 4 shows the energy harvesting properties of the RF-TENG. With the connection of commercial rectifier bridges and capacitors, RF-TENG is used to drive many low-power devices. Figure 4A shows the circuit diagram of a 7.8 cm × 4.8 cm RF-TENG powering low-power devices using a commercial capacitor under cyclic press and release conditions. Since the energy generated by RF-TENG is the form of AC power, it cannot directly power DC devices. A rectifier bridge and capacitor are introduced in the circuit for direct power supply. Low-power devices’ operating status is controlled by normally closed pushbutton switches. Figure 4B shows the charging capability of the RF-TENG for different capacitors. The commercial capacitors 0.47 μF, 1 μF, and 2.2 μF are charged to 4.1 V, 3.3 V, and 2.8 V within 40 s. The excellent energy harvesting properties of RF-TENG are verified. Figure 4C shows a schematic diagram of the RF-TENG driving a small calculator and watch operation and the curve during charging and discharging. The RF-TENG is charged to 2.92 V (Figure 4CⅠ), the normally closed pushbutton switch is clicked, and the low-power calculator and watch start to work (Video S1) and operate well (Figures 4CⅡ and 4CⅢ). In addition, the RF-TENG is capable of lighting up to 28 LEDs in series (Video S2), as shown in Figure 4D. Figure 4DⅠ shows the simple circuit diagram of RF-TENG driving LEDs. The 28 LEDs form the letters “RF” in series on the breadboard (Figure 4DⅡ) and are lighted by tapping the RF-TENG (Figure 4DⅢ). In summary, the excellent energy harvesting properties of RF-TENG show some prospects for commercial applications.

Video S1. RF-TENG for driving miniature calculators and watches, related to Figure 4

Video S2. RF-TENG for lighting up to 28 LEDS, related to Figure 4

### Dance sports and injury monitoring system

**Figure 5:**
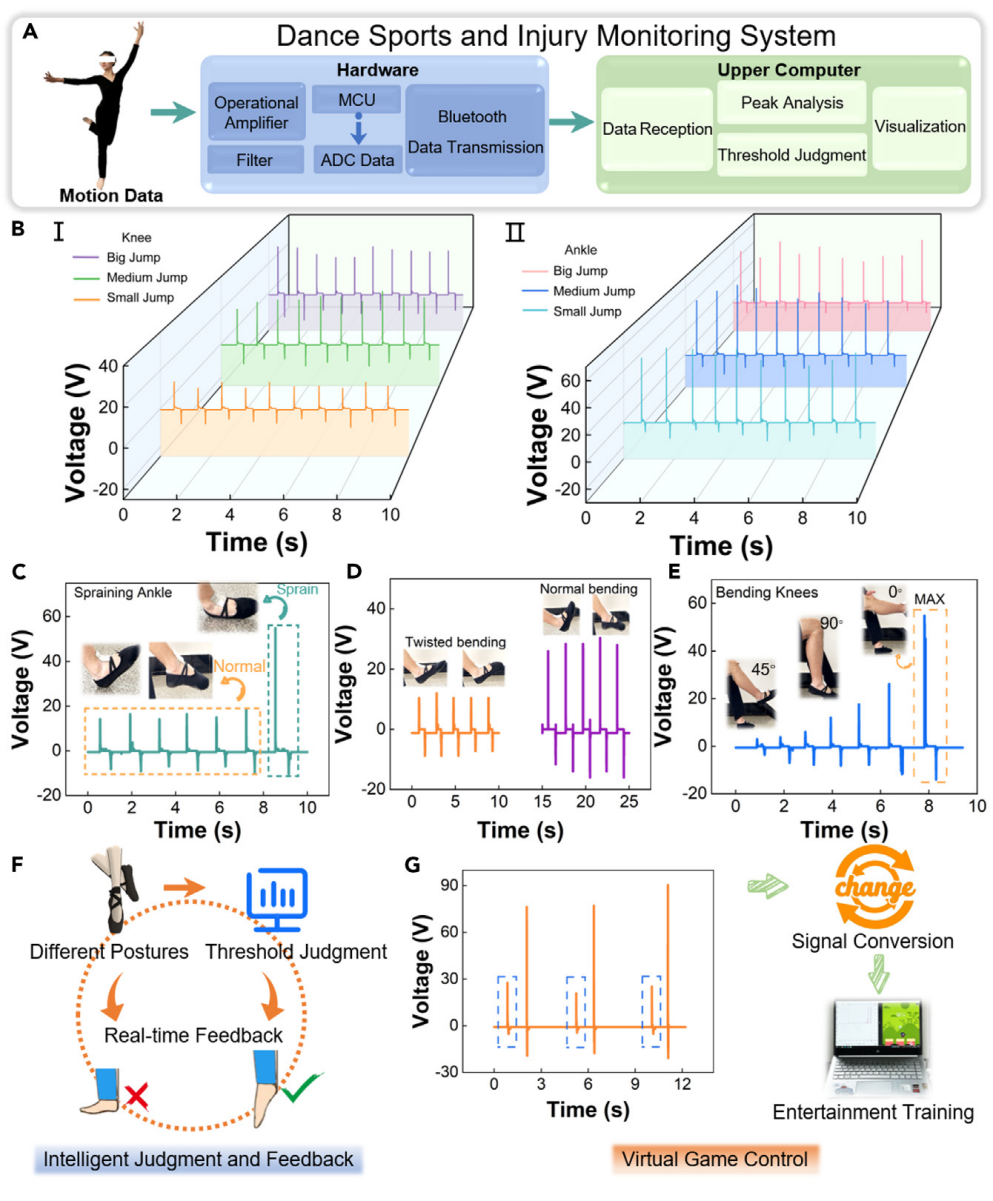
The application of DIMS (A) Schematic diagram of DIMS. (B) DIMS for dance ground-jumping techniques monitoring. (Ⅰ) The output voltage generated by the knee joint during dancers’ ground-jumping. (Ⅱ) The output voltage generated by the ankle joint during dancers’ ground-jumping. (C) DIMS for monitoring ankle sprains. (D) Output voltage of normal flexion and extension and post-injury flexion and extension of the ankle joint. (E) DIMS for monitoring knee hyperextension. (F) Flow diagram of DIMS for judging dance sports status. (G) Flow diagram of DIMS for virtual game control.

The ankle joint, as the distal major joint of the human body, is the foundation for the development of jumping quality. In dance ground-jumping techniques, the ankle joint plays an important supporting role. The knee and ankle joints are the foundation that affects the dancer’s ability to bounce, rotate, maintain the center of gravity, and support the dancer in doing various sports techniques, and are the guarantee for the dancer to master the dance techniques. Therefore, the RF-TENG is attached to the dancer’s knee and ankle joints for dance sports status monitoring. Figure 5 shows the application of the DIMS. Figure 5A shows the workflow of the DIMS. The system mainly consists of an RF-TENG sensor module, a data processing hardware module, and an upper computer intelligent analysis module. Considering that triboelectric sensors serve as high-impedance electronic devices, their generated voltage signal must be transformed to the analogue to digital converter (ADC) measurement range inside the microcontroller unit (MCU) through the operational amplifier and filter, then transmitted wirelessly via Bluetooth to the upper computer for virtual application and sports injury analysis. The knee and ankle joints play an important role in the aesthetics and coherence of the dancer’s sports during the dance ground-jumping techniques. From a mechanical perspective, the position of the knee and ankle joints during jumping must also be stable. Therefore, in the actual test process, dual sensing units are used to better monitor the lower limb power chain during dance sports and assist dancers in forming power stereotypes. Figure 5B shows the output voltage of the knee joint (Figure 5BⅠ) and ankle joint (Figure 5BⅡ) flexion and extension during big, medium, and small jumps in dance ground-jumping techniques. “Allegro” is the basic movement of classical ballet. According to the magnitude of the jump, it can be divided into small jump, medium jump, and big jump. Small jump is a basic form of jumping training, characterized by low height and fast speed. Medium jump is a larger take off based on a small jump pushing the ground, feeling the opposing force generated by gravity. Big jump is a movement where the powered leg is kicked up 90° or higher to generate stronger explosive force and take off.” The average voltage values generated by the knee joint during big, medium, and small jumps are 28.04 V, 26.72 V, and 12.36 V, respectively. The average voltage value generated in the knee joint during the small jump is much smaller than that of the medium and big jumps. This is because the small jump is the basic training method for dancers to master the rise and fall, focusing on training ankle strength and speed. The degree of plantar flexion of the dancer’s ankle joint can be determined based on the voltage. On the contrary, the average voltage values generated by the ankle joint during big, medium, and small jumps are 50.08 V, 52.72 V, and 51.88 V, respectively, which are relatively stable. This shows that the ankle joint maintains good stability during small, medium, and big jump training. The amplitude of the dancer’s movements is reflected by the output voltage generated by the knee and ankle joints. In addition, the interval time between peaks can also better reflect the dancer’s sports rhythm. The application of DIMS is beneficial for educators to objectively understand the basic laws of jumping movements, reduce blind training, and grasp the feasibility of training. Figure 5C shows the change in the output voltage of RF-TENG during the ankle sprain. RF-TENG is attached to the surface of the talus and fibula of the foot with Kinesio Tape, covering the anterior talofibular ligament. When the ankle joint is twisted, the area will undergo inversion movement. The sensor will produce a significant angle change, resulting in a sudden increase in voltage. However, in daily jumping sports, the sensor bending angle does not change significantly as a result of the load due to the different attachment positions. The average RF-TENG output voltage in normal flexion and extension is 15.77 V. After an ankle sprain, the RF-TENG output voltage is 54.8 V, showing a significant and sharp increase. Figure 5D shows the comparison of RF-TENG output voltage before and after the ankle sprain. The average output voltage generated by RF-TENG during normal ankle flexion and extension is 28.24 V, which is greater than the average output voltage of 10.72 V after sprain. The difference in output voltage value provides the possibility for the application of RF-TENG in sports injury prediction and rehabilitation status monitoring. Figure 5E shows the output voltage generated by the RF-TENG at different ranges of flexion and extension of the knee joint. The physiological flexion and extension angles of the knee joint are 135° and 0°, respectively. When the flexion angle is 45°, the average output voltage of RF-TENG is 4.26 V. When the flexion angle is 90°, the average output voltage of RF-TENG is 18.6 V. When the flexion angle is 135° (knee joint extension 0°), the range of knee joint flexion and extension reaches its maximum. The output voltage of RF-TENG is 54.8 V. When the knee joint exceeds this angle of movement, it is considered knee hyperextension. The change in the output voltage of RF-TENG provides a possibility for determining knee hyperextension. In summary, DIMS can not only be characterized to identify and predict sports injuries in the human knee and ankle joints but also provide a new method for monitoring rehabilitation status. In the previous tests, Kinesio tape (muscle patch) and skin film (bottom foam) are used to fix the sensor to ensure its comfort and stability when attached to the joints. In addition, the DIMS has real-time judgment and feedback functions to show and visualize the dancer’s movement status, as shown in Figure 5F. The sports data are transmitted in real-time by the RF-TENG sensor module to the upper computer intelligent analysis module, and the fast Fourier transform algorithm is applied to complete the recognition of the sports data. The degree of control of the dancer’s instep during sports is monitored by DIMS and the feedback results in the form of animations are presented by the upper computer intelligence analysis module (Video S3). Figure 5G shows the DIMS virtual game control function. As a front-end sensing unit, RF-TENG has the advantages of softness, portability, and lightness. The existence of data processing modules enables the upper computer intelligent analysis module to achieve game control. When the dancer jumps, the virtual game also jumps to avoid obstacles. The game character is controlled through signal sensing (Video S4). This adds an immersive experience for dancers in the dance training process. Meanwhile, the combination of feedback results in the form of animations improves the interest and efficiency of training. The design of DIMS promotes the intelligent application of flexible sensors in the field of sports assistance devices and provides new ideas for quantitative monitoring of dance techniques and sports injury prediction.

Video S3. DIMS for correct and incorrect dance sports status determination, related to Figure 5

Video S4. DIMS for virtual game control, related to Figure 5

### Dance technology recognition based on KNN algorithm

**Figure 6:**
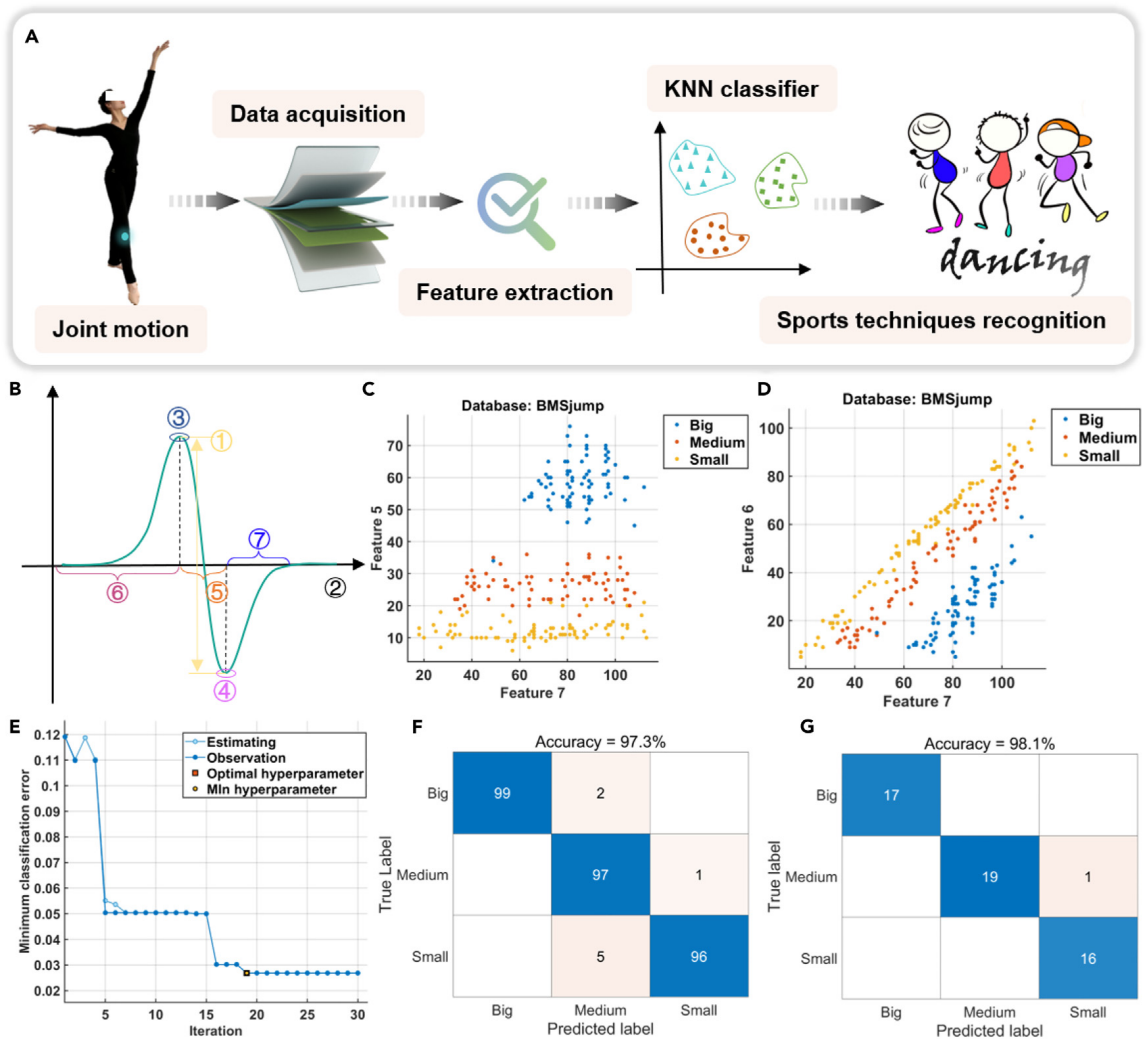
Recognition of dance ground-jumping techniques based on KNN algorithm (A) Flowchart for dance ground-jumping techniques recognition based on KNN algorithm. (B) Signal time-domain characteristic diagram. (C and D) Scatterplot of dance floor jumping techniques (small, medium, and big jumps). (E) Minimum classification error diagram. (F and G) Confusion matrix of training results and test results.

Considering the complexity of sensor signals when using naked-eye observation will affect the monitoring efficiency and accuracy, to monitor the voltage signals generated by RF-TENG during small, medium, and big jumps, machine learning techniques can be used to discover the regularity and difference of the signals from a big amount of characteristic parameter data. KNN is a commonly used machine learning algorithm for classification and regression problems. “K” stands for the number of nearest neighbors, for a new input sample, the K samples nearest to it are found by calculating its distance from all the samples in the training set and then the prediction is made based on the labels of these K samples. Figure 6A shows the recognition process of dance ground-jumping techniques based on KNN algorithm. The ankle signal characteristics did not change significantly during big, medium, and small jumps (Figure 5BⅡ), so the RF-TENG is attached to the knee position to acquire data. Then, features such as peaks, waveforms, etc. are extracted. Finally, KNN algorithm is applied to different dance ground-jumping techniques recognition. In the actual testing process, the big, medium, and small jump techniques are repeated several times and a total of 355 samples are obtained after preprocessing. The first 85% (300 samples) is the training set and the last 15% (53 samples, during the preprocessing process, two samples that did not meet the experimental conditions were removed) is the test set. Figure 6B shows seven time-domain features of the extracted signal after preprocessing. During the testing process, features ① ② ③ ④ cannot detect the regularity and differences of the signal. Features ⑤ ⑥ ⑦ represent the time difference between the lowest and highest points, the falling time, and the rising time, respectively, which can clearly distinguish the signal. Figures 6C and 6D show the scatterplot for three different dance ground-jumping techniques. According to feature ⑦ and feature ⑥, feature ⑦ and feature ⑤, it is not only possible to easily identify a dense arrangement of individual dance techniques of the same type, but it is also possible to clearly and efficiently distinguish clusters representing individual dance techniques from each other. Figure 6E shows the minimum classification error diagram, which is the process of using optimized KNN to train and optimize model parameters. The model optimization hyperparameters are: number of neighbors is 10, distance metric is Chebyshev, distance weight is inverse distance, and un-normalized data. Figures 6F and 6G show the confusion matrix for three different jumping techniques from the KNN classification results (K = 10). The accuracy of the training results is 97.3% and the accuracy of the test results is 98.1%. In addition, comparative information between KNN and different algorithms, neural networks, and other methods is provided (Table S2). The results show that the KNN algorithm has shown excellent performance in terms of training accuracy, testing accuracy, prediction speed, and training time. Our research findings provide a good solution for identifying complex human joint movements. The sensor can be used for techniques monitoring dance sports, showing its great potential for applications in areas such as sports assistance devices and sports monitoring.

### Conclusion

In summary, this work reported a recyclable flexible TENG-powered system for dance sports and injury monitoring. The dielectric layer consists of the PTFE film and wood fiber tissue encapsulated in PDMS, which can attach freely to the surface of the human knee and ankle joints to monitor low-frequency mechanical movements of the body due to their excellent flexibility and mechanical properties. With a response time of 17 ms and a recovery time of 26 ms, the RF-TENG significantly reduced data transfer times with the help of a dual sensing unit that better monitors the dancer’s lower limb movement. The RF-TENG also had excellent stability and durability, with a stable output voltage after 4,200 cycles and 30 days of resting. The DIMS based on RF-TENG module, a data processing hardware module, and an upper computer intelligence analysis module was successfully applied to monitor ankle sprains and knee hyperextension injuries. The computer intelligent analysis module realized virtual applications such as intelligent judgment and feedback on the dancer’s sports status and game control, which would be interesting during the boring training time. Combined with machine learning methods, three different classes of sports techniques were successfully recognized and classified with training and testing accuracies of 97.3% and 98.1%, respectively. The developed DIMS based on the recyclable flexible TENG-powered system provides a viable platform for flexible sensor applications in the field of sports assistance and intelligent motion.

### Limitations of the study

This study developed a DIMS, and applied it to monitor the dance techniques. Nevertheless, two limitations must be noted. Firstly, the size and transmission speed of the hardware module need to be further optimized to minimize motion interference. Secondly, the visualization content of the upper computer intelligent analysis module needs to be expanded to enrich the motion scene.

## STAR★Methods

### Key resources table

**Table:** 

REAGENT or RESOURCE	SOURCE	IDENTIFIER
**Chemicals, peptides, and recombinant proteins**		

SYLGARD 184	Shenzhen Huazhisheng New Materials Technology Co., Ltd. (Guangdong, China)	N/A
PTFE film	Taizhou Yongchen New Materials Co., Ltd. (Jiangsu, China)	N/A
Al foil	Dongguan Xinzhan Packaging Products Co., Ltd. (Dongguan, China)	N/A
Enameled wire	Suzhou Wujiang Shenzhou Bimetal Cable Co., Ltd. (Jiangsu, China)	N/A

**Software and algorithms**		

Origin 2022	OriginLab	N/A
Autodesk 3ds Max	Autodesk	N/A
COMSOL Multiphysics 6.0	COMSOL	N/A
Adobe Illustrator 2020	Adobe	N/A

### Resource availability

#### Lead contact

Yupeng Mao (maoyupeng@pe.neu.edu.cn).

#### Materials availability

Materials used in the study are commercially available.

#### Data and code availability

All data reported in this paper will be shared by the lead contact upon reasonable request.

No new code was generated during the course of this study.

Any additional information required to reanalyze the data reported in this paper is available from the lead contact upon reasonable request.

### Method details

#### Materials

The basic components and curing agent of SYLGARD 184 were purchased from Shenzhen Huazhisheng New Materials Technology Co., Ltd. (Guangdong, China). Aluminum foil was purchased from Dongguan Xinzhan Packaging Products Co., Ltd. (Dongguan, China). PTFE film was purchased from Taizhou Yongchen New Materials Co., Ltd. (Jiangsu, China). The enameled wire was purchased from Suzhou Wujiang Shenzhou Bimetal Cable Co., Ltd. (Jiangsu, China). All materials were used as is, without purification.

#### Preparation of disposable tissue dielectric layer

In this work, wood fiber material tissue was chosen as the dielectric layer. The preparation process was as follows: Firstly, the wood fiber tissues were soaked in anhydrous ethanol for 10 minutes for cleaning. Secondly, the cleaned wood fiber tissues were laid flat on the FEP substrate and heated on a heating table at a temperature of 80°C for 15 min until dry. Finally, the dried wood fiber tissues were trimmed properly and the dielectric layer preparation was completed.

#### Preparation of PDMS film encapsulation layer and support layer

Firstly, the basic components and curing agent of SYLGARD 184 were mixed in a 10:1 mass ratio and stirred for 5 minutes until completely mixed. Secondly, the bubbles generated after mixing were cleaned with an ultrasonic cleaner for 10 minutes to remove the bubbles. Finally, the PDMS film was prepared by pouring it into the mold and curing it in the oven at 80°C for 15 min.

#### Preparation of RF-TENG sensor

Firstly, the aluminum foil was attached to the wood fiber tissue and PTFE film respectively. The enamelled wire was led out by the aluminum foil. Secondly, the wood fiber tissue and PTFE film were attached to the PDMS support layer. Finally, PDMS film was used as an encapsulation layer to wrap the entire sensor. The optical diagram of RF-TENG is shown in Figure S8.

#### COMSOL multiphysics

To determine the working mechanism of the RF-TENG, a finite element method simulation using COMSOL Multiphysics was constructed to evaluate the electrical potential distribution between the layers of PTFE and Tissue. The thickness of both Tissue and PTFE is 1mm, and the length is 78mm. The relative dielectric constants of PTFE and Tissue are 2 and 4, respectively. Simulate sensor operation by changing the distance between layers.

#### Characterizations

The electrical properties of RF-TENG were tested by oscilloscopes (sto1102c, micsig), as shown in Figure S9. The finite element simulation of the working mechanism of RF-TENG was performed by COMSOL Multiphysics software. The test equipment includes linear motors used to simulate the movement of human joints.

#### Composition and workflow of the data processing module

The hardware module consists of a transmitter side and a receiver side (Figure S10). The transmitter side includes a signal processing circuit, STM32 master controller and Bluetooth host module. The receiver side includes a Bluetooth slave module and CH340 chip. The generated voltage signal is transformed by an operational amplifier and filtered into the measurement range of the ADC of the MCU. MCU reads the data collected by the internal ADC and transmits it wirelessly to the upper computer via the Bluetooth module for data analysis. The circuit diagram is shown in Figure S11. The maximum transmission distance of the data processing module was tested, as shown in Figure S12. In open spaces, 65m ultra-long distance data transmission can be achieved.
