# Supplementary material for: Machine learning-assisted novel recyclable flexible triboelectric nanogenerators for intelligent motion
Source: iScience. 2024 Mar 29;27(4):109615. doi: 10.1016/j.isci.2024.109615 (PMC11022051; doi:10.1016/j.isci.2024.109615)
Supplement: Document S1. Figures S1–S12 and Tables S1 and S2 [file mmc1.pdf]

iScience, Volume *27*

Supplemental information

Machine learning-assisted novel recyclable flexible triboelectric nanogenerators for intelligent motion

Yuzhang Wen, Fengxin Sun, Zhenning Xie, Mengqi Zhang, Zida An, Bing Liu, Yuning Sun, Fei Wang, and Yupeng Mao

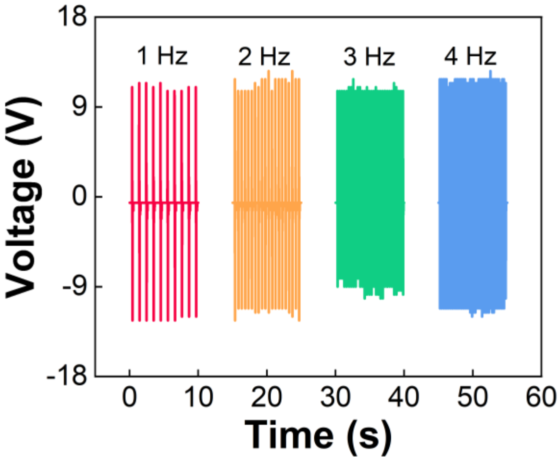

Figure S1. RF-TENG output voltage at different frequencies, related to Figure 3.

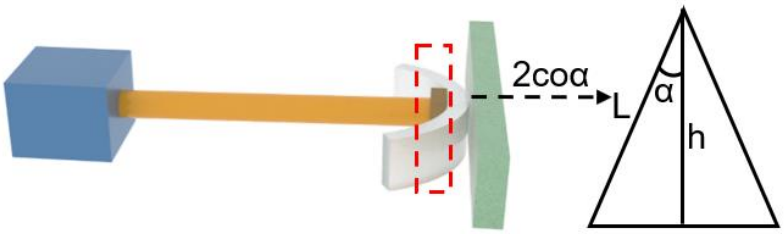

Figure S2. Schematic diagram of linear motor simulation angle test, related to Figure 3.

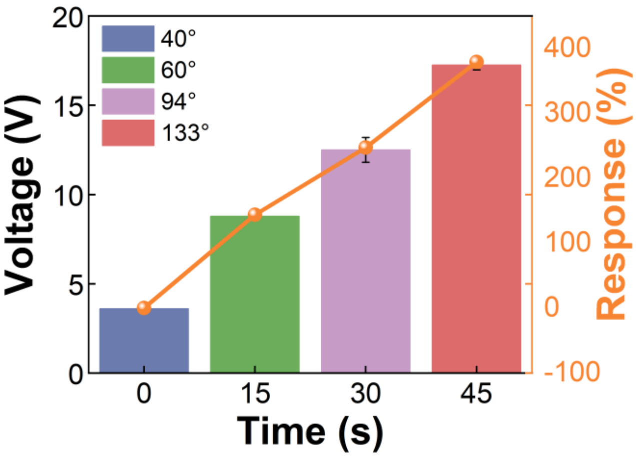

Figure S3. RF-TENG output voltage and response at different angles, related to Figure 3.

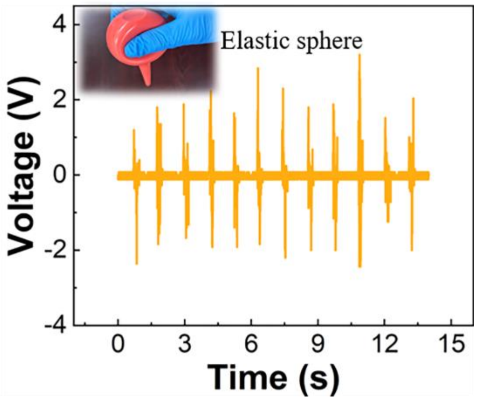

Figure S4. Voltage signals of the RF-TENG generated by an elastic sphere, related to Figure 3.

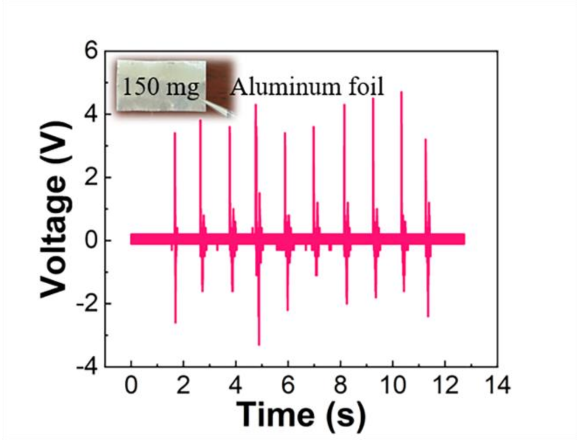

Figure S5. Voltage signals of the RF-TENG generated by an aluminum foil with 150 mg, related to Figure 3.

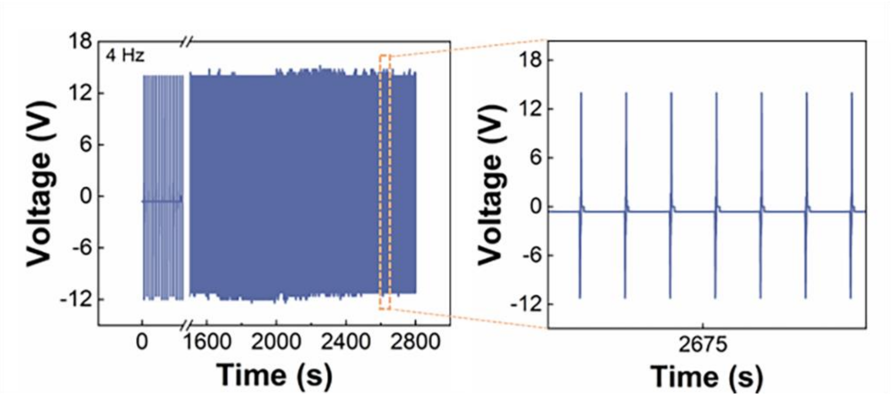

Figure S6. Output voltage of RF-TENG after 4200 cycles and its detail, related to Figure 3.

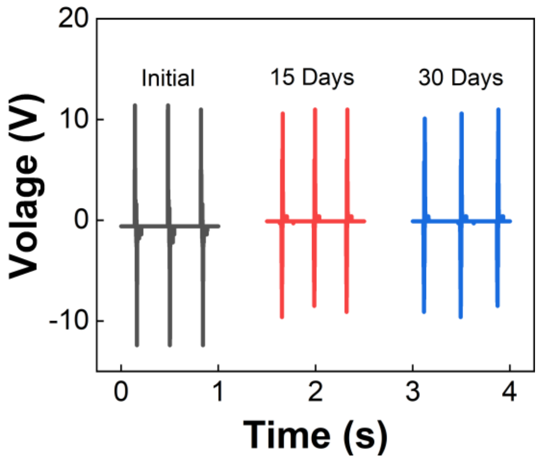

Figure S7. RF-TENG output voltage after initial, 15 days and 30 days of resting condition, related to Figure 3.

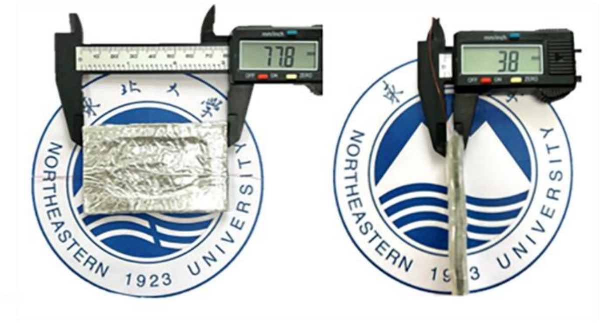

Figure S8. The optical diagram of RF-TENG, related to STAR Methods.

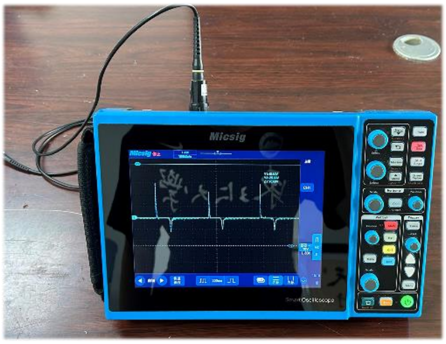

Figure S9. The optical diagram of oscilloscopes, related to STAR Methods.

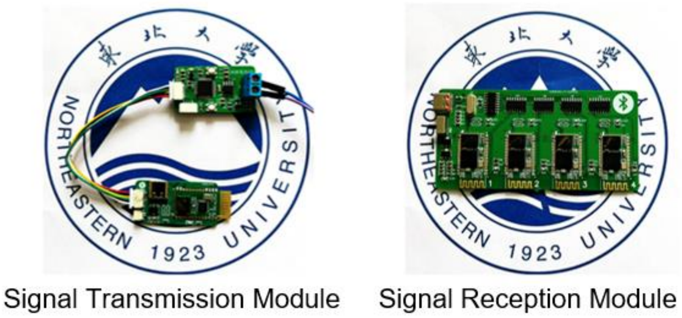

Figure S10. Optical diagram of the data processing hardware modules, related to STAR Methods.

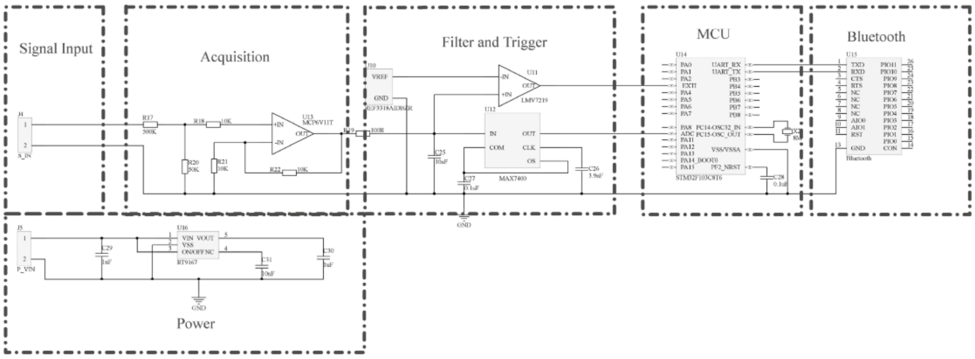

Figure S11. Circuit diagram of data processing hardware module,related to STAR Methods.

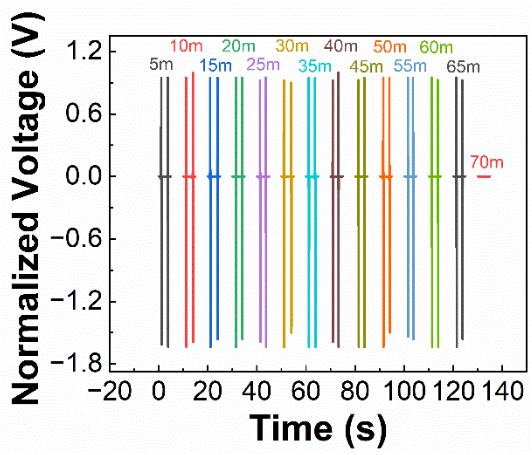

Figure S12. Testing the transmission distance of data processing module, related to STAR Methods.

Table S1. Comparison between RF-TENG fast response time with other works, related to Figure 3.

Article	Material	Mechanism	Time
Strong and flame-retardant wood-based triboelectric nanogenerators toward self-powered building fire protection	Wood, PTFE	Single electrode mode	50 ms[1]
A flexible, stretchable and triboelectric smart sensor based on graphene oxide and polyacrylamide hydrogel for high precision gait recognition in Parkinsonian and hemiplegic patients	Sliver nanowire, GO-PAM hydrogel film	Contact separation mode	500 ms[2]
Anatomically Designed Triboelectric Wristbands with Adaptive Accelerated Learning for Human–Machine Interfaces	Natural latex, PTFE	Single electrode mode	100 ms[3]
Nano-fiber based self-powered flexible vibration sensor for rail fasteners tightness safety detection	Silicone rubber, PVDF/BTO nanofibers	Contact separation mode	40 ms[4]
Highly tough and conductive hydrogel based on defect-patched reduction graphene oxide for high-performance self-powered flexible sensing micro-system	rGO-CMOF/DNH	N/A	40 ms[5]
Continuous fabrication of core-sheath fiber for strain sensing and self-powered application	SBS/MWCNTs	N/A	50ms[6]
Deep learning assisted ternary electrification layered triboelectric membrane sensor for self-powered home security	Kapton/Cu	Freestanding Triboelectric Layer Mode	320ms[7]
**This work**	Tissue, PTFE	Contact separation mode	17 ms

[1]. Luo, J., Shi, X., Chen, P., Han, K., Li, X., Cao, X., and Wang, Z.L. (2022). Strong and flame-retardant wood-based triboelectric nanogenerators toward self-powered building fire protection. Mater Today Phys 27. 10.1016/j.mtphys.2022.100798
[2]. Wang, Z., Bu, M., Xiu, K., Sun, J., Hu, N., Zhao, L., Gao, L., Kong, F., Zhu, H., Song, J., et al. (2022). A flexible, stretchable and triboelectric smart sensor based on graphene oxide and polyacrylamide hydrogel for high precision gait recognition in Parkinsonian and hemiplegic patients. Nano Energy 104. 10.1016/j.nanoen.2022.107978.
[3]. Fang, H., Wang, L., Fu, Z., Xu, L., Guo, W., Huang, J., Wang, Z.L., and Wu, H. (2023). Anatomically Designed Triboelectric Wristbands with Adaptive Accelerated Learning for Human-Machine Interfaces. Adv Sci 10. 10.1002/advs.202205960.
[4]. Meng, Y., Yang, J., Liu, S., Xu, W., Chen, G., Niu, Z., Wang, M., Deng, T., Qin, Y., Han, M., et al. (2022). Nano-fiber based self-powered flexible vibration sensor for rail fasteners tightness safety detection. Nano Energy 102. 10.1016/j.nanoen.2022.107667.
[5]. Yue, J., Li, C., Ji, X., Tao, Y., Lu, J., Cheng, Y., Du, J., and Wang, H. (2023). Highly tough and conductive hydrogel based on defect-patched reduction graphene oxide for high-performance self-powered flexible sensing micro-system. Chem Eng J 466. 10.1016/j.cej.2023.143358.
[6]. Zhong, J., Chen, R., Shan, T., Peng, F., Qiu, M., Sun, Z., Ren, K., Ning, C., Dai, K., Zheng, G., et al. (2023). Continuous fabrication of core-sheath fiber for strain sensing and self-powered application. Nano Energy 118. 10.1016/j.nanoen.2023.108950.
[7]. Xu, J., Yin, J., Fang, Y., Xiao, X., Zou, Y., Wang, S., and Chen, J. (2023). Deep learning assisted ternary electrification layered triboelectric membrane sensor for self-powered home security. Nano Energy 113. 10.1016/j.nanoen.2023.108524.

Table S2. Multidimensional comparison table between KNN and other methods, related to Figure 6.

	**SVM**	**NB**	**MLP**	**CNN**	**Our methods**
**Training accuracy**	97.30%	97.30%	95.70%	97.00%	97.30%
**Testing accuracy**	88.70%	94.30%	96.20%	90.60%	98.10%
**Prediction speed**	2200obs/sec	3100obs/sec	2900obs/sec	1500obs/sec	4000obs/sec
**Training time**	2.96s	3.08s	4.69s	5.44s	2.48s
